# Regional Variation in Gut Microbial Community Structure and Predicted Phenotypic Characteristics of Farmed Large Yellow Croaker (*Larimichthys crocea*)

**DOI:** 10.3390/biology15171463

**Published:** 2026-08-27

**Authors:** Ling Lin, Wangyang Jin, Huijuan Wang, Xiaojun Yan, Lihua Jiang, Shun Chen

**Affiliations:** 1 Pingyang Institute of Science and Technology Innovation, Wenzhou 325400, China; 13587985005@163.com (L.L.); jwy17805805806@outlook.com (W.J.); wang_hui_j@163.com (H.W.); yanxj@zjou.edu.cn (X.Y.); 2 Pingyang Branch of Wenzhou Ecological Environment Bureau, Environmental Protection Building, No. 269 Huishuihe Road, Kunyang Town, Pingyang County, Wenzhou 325400, China; 3 National Engineering Research Center for Marine Aquaculture, Zhejiang Ocean University, Zhoushan 316022, China

**Keywords:** 16S rRNA, large yellow croaker, gut microbial community

## Abstract

Large yellow croaker *(Larimichthys crocea*) is an economically important marine fish species in China, yet regional variation in its gut microbial community structure and functional characteristics remains insufficiently understood. This study aimed to characterize and compare the gut microbiota of farmed large yellow croaker across five representative aquaculture regions. Intestinal microbial communities were characterized using 16S rRNA gene sequencing, followed by analyses of microbial diversity, taxonomic composition, predicted bacterial phenotypes, co-occurrence networks, and environmental associations. The five cultured populations shared a broadly similar microbial community structure dominated by *Proteobacteria*, *Bacteroidota*, and *Firmicutes*, whereas the relative abundance of several dominant taxa varied among regions. Alpha-diversity differences were mainly associated with community evenness and dominance rather than estimated richness. Although regional samples partially overlapped in ordination analyses, the aquaculture region explained 41.4% of the variation in gut microbial composition, indicating significant region-associated differentiation. Predicted microbial characteristics were comparatively similar among cultured populations, despite variation in taxonomic composition, while microbial network organization also differed across regions. Environmental variables showed clearer associations with water microbiota than with gut microbiota, and local aquaculture water represented a detectable but limited potential source of intestinal microorganisms. Overall, farmed large yellow croaker maintained a shared gut microbial community structure but exhibited region-associated variation in community composition and predicted bacterial phenotypes. These findings provide a regional baseline for understanding gut microbial variation in farmed large yellow croaker under different aquaculture conditions.

## Simple Summary

Large yellow croaker is an important farmed marine fish in China, but little is known about how its gut microorganisms vary among farming regions. In this study, we compared the gut microbiota of fish collected from five major aquaculture areas. The fish shared a similar core microbial community, mainly consisting of *Proteobacteria*, *Bacteroidota*, and *Firmicutes*. However, the relative abundance of some bacterial groups and the organization of microbial interaction networks differed among regions. The predicted microbial functions were generally similar, suggesting that different bacterial communities may perform comparable roles in the gut. Water conditions were more closely related to microorganisms in the surrounding water than to those in the fish intestine, and aquaculture water contributed only a small proportion of gut microorganisms. These results show that farmed large yellow croaker maintains a relatively stable gut microbial community structure while still exhibiting regional differences. This study provides baseline information for future research on fish health and region-specific aquaculture management.

## 1. Introduction

Large yellow croaker (*Larimichthys crocea*) is one of the most economically important marine fish species in Chinese mariculture. Over the past few decades, cage culture of this species has expanded rapidly along the southeastern coast of China [1,2,3]. According to the 2020 China Fisheries Yearbook, the total production of marine cage-cultured large yellow croaker exceeds 225,000 tons, generating substantial commercial value [4,5]. The fish gut microbiota is closely associated with host health and performs important functions in nutrient metabolism, immune regulation, intestinal development, and environmental adaptation [6,7,8,9,10]. Therefore, understanding the composition and ecological characteristics of gut microbial communities is important for improving aquaculture management and maintaining fish health, particularly under increasing environmental pressures associated with intensive aquaculture and climate change.

From a microecological perspective, the fish gut microbiota can be viewed as a local host-associated microbial community embedded within a larger environmental microbial metacommunity. Community composition may consequently reflect the combined effects of microbial availability in the surrounding source pool, dispersal or exposure from the external environment, local environmental conditions, and selection within the host-associated habitat [11,12,13]. In aquatic animals, continuous contact with the surrounding water provides opportunities for microbial exchange, but the gut community does not simply reproduce the environmental microbiota because only a subset of environmentally available microorganisms may persist within the intestinal habitat [12,13]. Thus, regional variation in fish gut microbiota may arise not only from differences in the environmental microbial source pool, but also from differences in the ecological conditions under which microorganisms are exposed to and maintained within the host. Previous studies of large yellow croaker and other fishes have demonstrated that gut microbial communities can vary with diet, developmental stage, disease status, habitat, domestication, and environmental stress [6,14,15,16,17,18,19,20]. Nevertheless, most studies have examined host-associated or environmental microbiota separately, and relatively little is known about how regional environmental heterogeneity, surrounding water microbial communities, and gut microbial patterns are linked across aquaculture regions.

This knowledge gap is particularly relevant to large yellow croaker because the species is cultured across coastal and island-associated areas with contrasting environmental and farming conditions. Salinity, dissolved oxygen, nutrient availability, water exchange, hydrodynamic conditions, and culture facilities can vary among regions and may influence both the composition of environmental microbial source pools and the exposure of fish to these microorganisms [1,21,22]. Culture settings such as cage and fish-raft systems may further differ in water flow, organic matter accumulation, microbial exposure, and local management conditions [23,24]. Under such conditions, comparisons among geographically separated farming regions provide an opportunity to determine whether gut microbial communities exhibit reproducible regional signatures and whether these patterns covary with surrounding water microbiota and measured environmental conditions. Importantly, such associations do not by themselves identify the mechanisms of microbial community assembly, because host-related factors, diet, management practices, and unmeasured microbial sources may also contribute to the observed variation.

To address these knowledge gaps, the present study collected intestinal and water samples from five representative aquaculture regions, together with one wild reference group from the Nanji Archipelago. These sites represented different regional aquaculture settings under real production conditions, mainly including cage culture and fish-raft culture systems. Using full-length 16S rRNA gene sequencing, we characterized regional variation in gut microbial diversity and composition and evaluated its associations with surrounding water microbial communities and measured environmental factors. Source attribution and co-occurrence analyses were further used as complementary approaches to assess the potential contribution of water-associated microorganisms and to describe regional microbial association patterns. By integrating host-associated and environmental microbial data within a regional microecological framework, this study aims to improve understanding of spatial variation in the gut microbiota of large yellow croaker and to provide an ecological basis for region-specific aquaculture health management.

## 2. Materials and Methods

### 2.1. Fish Tissues and Water Sampling

**Figure 1:**
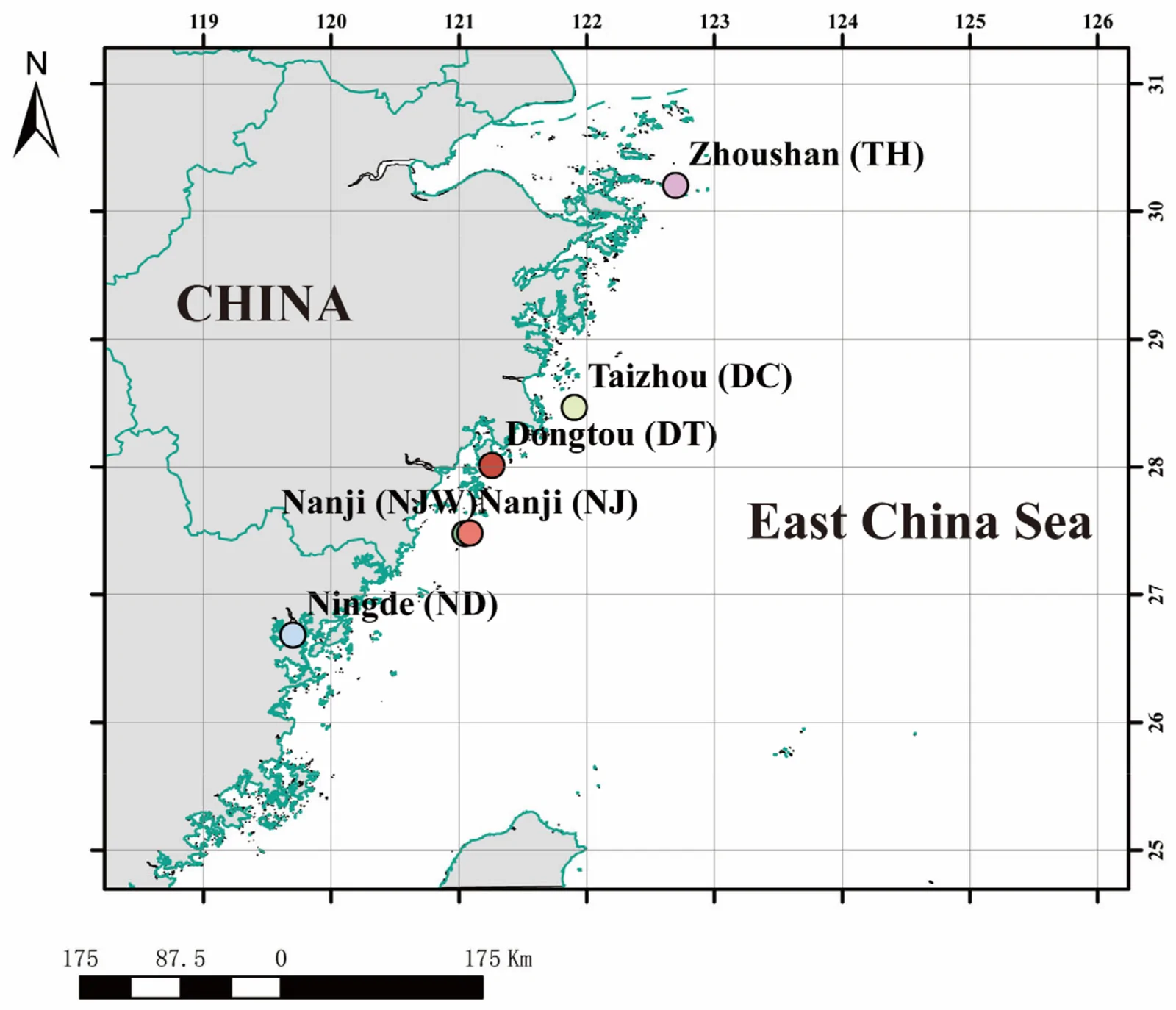
Sampling sites included Ningde (ND), Nanji wild (NJW), Nanji cultured (NJ), Dongtou (DT), Taizhou (DC), and Zhoushan (TH). Colored circles indicate the locations of the sampling points.

*Larimichthys crocea* specimens were collected from five representative locations in the East China Sea, including Zhoushan (TH), Taizhou (DC), and the Dongtou (DT) and Nanji (NJ and NJW) archipelagos in Wenzhou, all within Zhejiang Province, as well as Ningde (ND) in Fujian Province. The sampling locations are shown in Figure 1, and the environmental parameters of each sampling region are summarized in Supplementary Table S1. These sites were selected to represent distinct aquaculture systems and environmental settings: Zhoushan corresponds to enclosure-based aquaculture; Dachen Island (Taizhou, DC), Dongtou Island, and the Nanji Archipelago correspond to large offshore deep-water cage culture; and Ningde represents high-density raft aquaculture in a semi-enclosed bay. All samples were collected in September to reduce seasonal variation. During site selection, farmed populations with comparable stocking densities and similar feeding practices, including the use of similar commercial feeds, were preferentially selected to minimize the influence of major management-related differences. Most aquacultured populations were reared in cage culture systems, whereas the ND group was collected from a fish-raft culture system. In addition, wild individuals (NJW) were captured from the Nanji Archipelago and used as a natural reference group for comparison with aquacultured populations. In total, intestinal samples were collected from 15 aquacultured large yellow croakers from each aquaculture region and from 9 wild individuals from the Nanji Archipelago. For each aquaculture region, intestinal tissues from 15 fish with similar body size were collected, and samples from every three individuals were pooled to generate one composite intestinal sample, resulting in five replicated composite samples per aquaculture region. This pooling strategy was adopted to obtain more representative population-level gut microbial profiles for each aquaculture region and to reduce the influence of stochastic inter-individual variation. In contrast, the wild individuals were opportunistically obtained and were limited in number. If the same pooling strategy had been applied to the wild fish, only three composite samples could have been generated, which would have further reduced the biological information available from this limited wild population. Therefore, the nine wild fish were analyzed individually. Intestinal tissue samples were aseptically dissected, gently rinsed with sterile physiological saline to remove digesta, and immediately frozen in liquid nitrogen for DNA extraction. Only the rinsed intestinal tissues, rather than the intestinal contents, were used for subsequent DNA extraction. (The intestines of the 9 wild croakers were individually stored in 9 cryotubes.) After collection, all samples were immediately frozen in liquid nitrogen and shipped in dry ice boxes to the sequencing company for sequencing. The biometric characteristics of the large yellow croakers and detailed information on the sampling design for each group are provided in Supplementary Table S1.

Bacterial samples from large yellow croaker aquaculture waters were collected by vacuum filtration, using a 0.22 μm pore-size filter to process 1.5 L of water. This method produced filter samples enriched with aquaculture water microbes. These samples were immediately placed into cryotubes, preserved in liquid nitrogen, and then transported in dry ice boxes to a sequencing facility for microbial sequencing analysis.

### 2.2. Microbiome Analysis

#### 2.2.1. DNA Extraction, PCR Amplification, and PacBio Sequencing

Bacterial DNA was extracted from intestinal tissues using the Power Soil^®^ DNA Isolation Kit (MoBio, Carlsbad, CA, USA) following the manufacturer’s instructions. The full-length 16S rDNA gene was amplified using the forward primer 27F (5′-AGRGTTTGATYNTGGCTCAG-3′) and reverse primer 1492R (5′-TASGGHTACCTTGTTASGACTT-3′). PCR reactions were performed in a 30 μL system containing 1.5 μL of DNA template, 15 μL of KOD One™ PCR Master Mix (TOYOBO, Osaka, Japan), and 1.8 μL of each primer. The thermal cycling conditions were as follows: initial denaturation at 95 °C for 2 min; followed by 25 cycles of denaturation at 98 °C for 10 s, annealing at 55 °C for 30 s, and extension at 72 °C for 90 s; and a final extension at 72 °C for 2 min. PCR products were checked by electrophoresis on a 1.8% agarose gel and purified using VAHTS DNA Clean Beads (Vazyme, Nanjing, China) prior to sequencing. Purified PCR products were used to construct the sequencing library on the PacBio platform (Biomarker Technologies Company, Beijing, China [25]) according to the manufacturer’s specifications. To minimize contamination, sterile disposable instruments and gloves were used during sample collection. Sterile disposable instruments and gloves were used during sample collection to minimize potential contamination. DNA extraction blanks and PCR no-template controls were included as negative controls during laboratory processing. No detectable amplification was observed in the negative controls, and they were therefore not included in the final PacBio sequencing library.

#### 2.2.2. Sequence Processing and Microbial Diversity Analysis

Raw PacBio subreads were processed using SMRT Link (version 8.0) to generate circular consensus sequencing (CCS) reads. Barcode sequences were identified and samples were demultiplexed using Lima (version 1.7.0). Primer sequences and sequences outside the specified quality and length criteria were removed, and chimeric sequences were identified and excluded using UCHIME (version 8.1). High-quality sequences were clustered into operational taxonomic units (OTUs) at 97% sequence similarity using USEARCH (version 10.0). Taxonomic annotation was performed against the SILVA 138 reference database using classify-consensus-blast, followed by the naïve Bayes-based classify-sklearn method for sequences that could not be precisely assigned, as implemented in the QIIME 2 q2-feature-classifier plugin (version 2020.6).To minimize the influence of differences in sequencing depth on community diversity estimates, samples were rarefied to a common sequencing depth before diversity analyses. Alpha-diversity indices, including Shannon, Chao1, Simpson, and Pielou indices, were calculated using QIIME2 (version 2020.6). Beta diversity was evaluated using Bray–Curtis dissimilarities, and community patterns were visualized by principal coordinate analysis (PCoA) and non-metric multidimensional scaling (NMDS). Differences in microbial community composition among groups were assessed by permutational multivariate analysis of variance (PERMANOVA) using the ‘adonis’ function in the R package ‘vegan’ with 999 permutations [26]. Statistical analyses and visualization were conducted using QIIME2 and R (version 4.2.1), as appropriate.

#### 2.2.3. Functional Prediction and Co-Occurrence Network Analysis

Predicted microbial phenotypes, including aerobic and anaerobic characteristics, Gram status, biofilm formation, pathogenic potential, mobile-element content, and oxidative-stress tolerance, were predicted using BugBase.

Microbial co-occurrence networks were constructed independently for each regional group based on Spearman rank correlations among OTUs. Pairwise associations were subjected to Benjamini–Hochberg correction for multiple testing, and only associations with an absolute Spearman correlation coefficient of |ρ| ≥ 0.60 and an FDR-adjusted *p* < 0.05 were retained as network edges. Both positive and negative associations meeting these criteria were retained. The same correlation metric and filtering thresholds were applied consistently to all regional groups to ensure methodological comparability among networks. The retained associations were assembled into undirected networks using the ‘igraph’ package in R (version 4.4.1), and isolated nodes with a degree of zero were removed before subsequent topological analyses. Networks were visualized and modular structures were characterized in Gephi (version 0.11.2) [27]. For the Zi–Pi analysis, the adjacency matrix was converted to a binary presence–absence matrix, and the within-module connectivity (Zi) and among-module connectivity (Pi) were calculated for each node. Nodes were classified as peripherals (Zi < 2.5, Pi < 0.62), connectors (Zi < 2.5, Pi > 0.62), module hubs (Zi > 2.5, Pi < 0.62), or network hubs (Zi > 2.5, Pi > 0.62) [28,29]. These networks were interpreted as exploratory microbial co-occurrence patterns rather than as direct evidence of biological interactions.

#### 2.2.4. Microbial Source Tracking

SourceTracker was used to assess the potential contribution of surrounding water microbial communities to the intestinal microbiota of *L. crocea* [30]. Water microbiota collected from each aquaculture region were treated as potential source communities, whereas fish gut microbiota were treated as sinks. Source contributions and the unassigned fraction were estimated using the SourceTracker framework. Because surrounding water was the only systematically sampled environmental microbial source in the present study, the analysis was used as an exploratory assessment of water-associated microbial contributions rather than a comprehensive reconstruction of all potential microbial sources.

#### 2.2.5. Environmental Association Analysis

Canonical correspondence analysis (CCA) was performed to evaluate associations between measured environmental variables and microbial community composition. Candidate environmental variables included salinity, pH, dissolved oxygen (DO), water temperature (WT), total nitrogen (TN), nitrate (NO_3_^−^), nitrite (NO_2_^−^), ammonium (NH_4_^+^), total phosphorus (TP), and phosphate (PO_4_^3−^). These variables represented physicochemical parameters measured consistently across sampling regions.

Environmental variables were standardized before ordination analysis. Multicollinearity was evaluated using variance inflation factors (VIFs), and variables with VIF values >10 were sequentially removed until an acceptable level of collinearity was achieved. The statistical significance of the final CCA model, constrained axes, and individual environmental terms was assessed using 999 permutation tests. CCA was used to evaluate statistical associations between microbial community variation and measured environmental conditions rather than to infer direct causal effects.

## 3. Results

### 3.1. OTU Data Processing and Analysis

After sequencing all samples and identifying them via barcodes, a total of 1,954,505 CCS (Circular Consensus Sequencing) reads were obtained. Each sample generated at least 41,402 CCS reads, with an average of 57,485 CCS reads per sample. Using the SILVA database as a reference, feature sequences were taxonomically annotated through a combination of the Naive Bayesian classifier and alignment methods, allowing the identification of species classification information for each feature. Subsequently, the community composition of each sample at various taxonomic levels (phylum, class, order, family, genus, species) was analysed. The QIIME software was used to generate species abundance tables at different taxonomic levels, and R language tools were employed to visualize the microbial community structure at each taxonomic level across the samples.

### 3.2. Alpha and Beta Diversity Analysis

**Figure 2:**
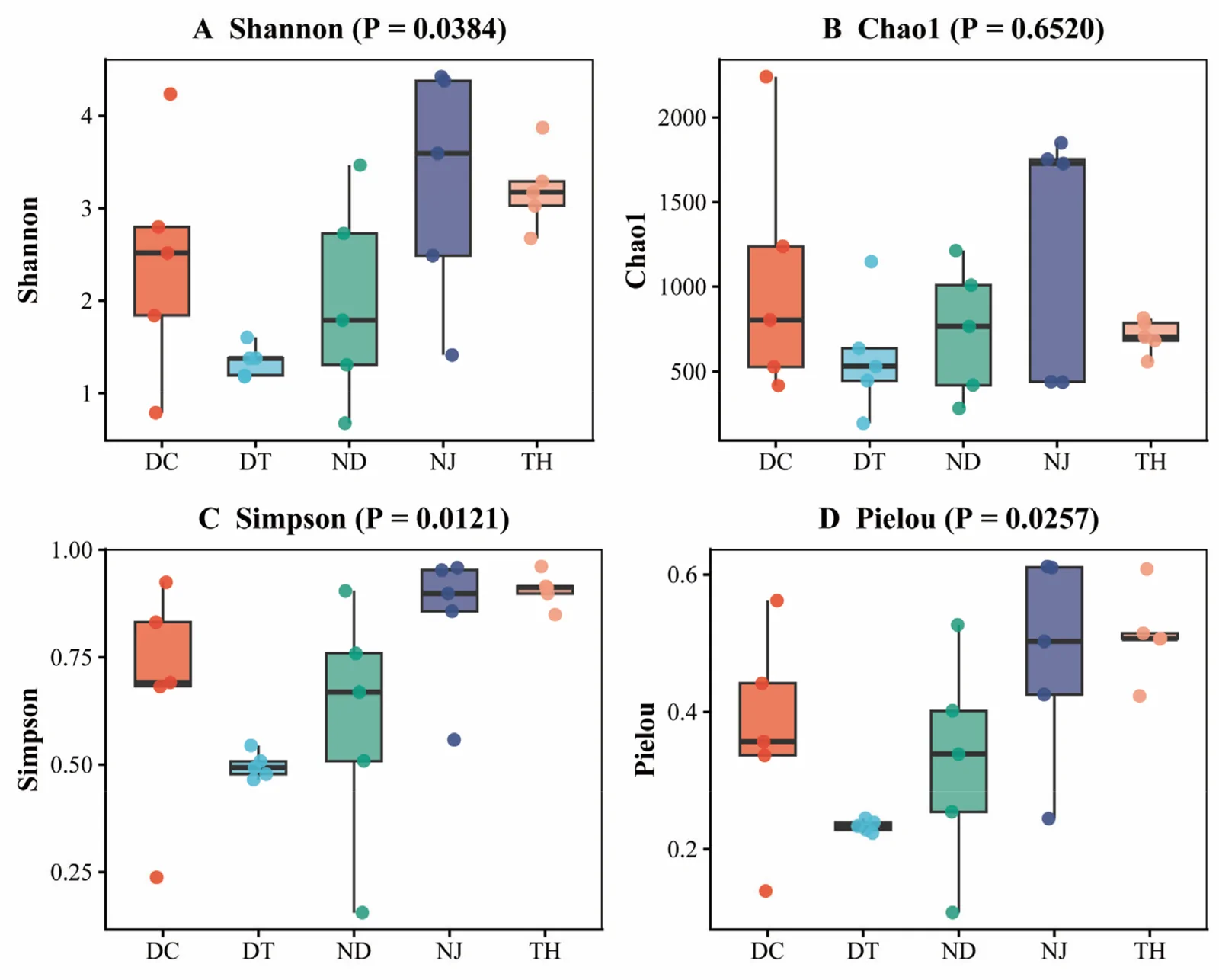
Alpha diversity of gut microbial communities among the five aquaculture regions. (**A**) Shannon diversity index. (**B**) Chao1 richness index. (**C**) Simpson index. (**D**) Pielou’s evenness index. Boxplots show the distribution of alpha-diversity values for composite intestinal samples from each aquaculture region, and points represent individual composite samples (*n* = 5 composite intestinal samples per region). Statistical differences among the five aquaculture regions were tested using the Kruskal–Wallis test.

**Figure 3:**
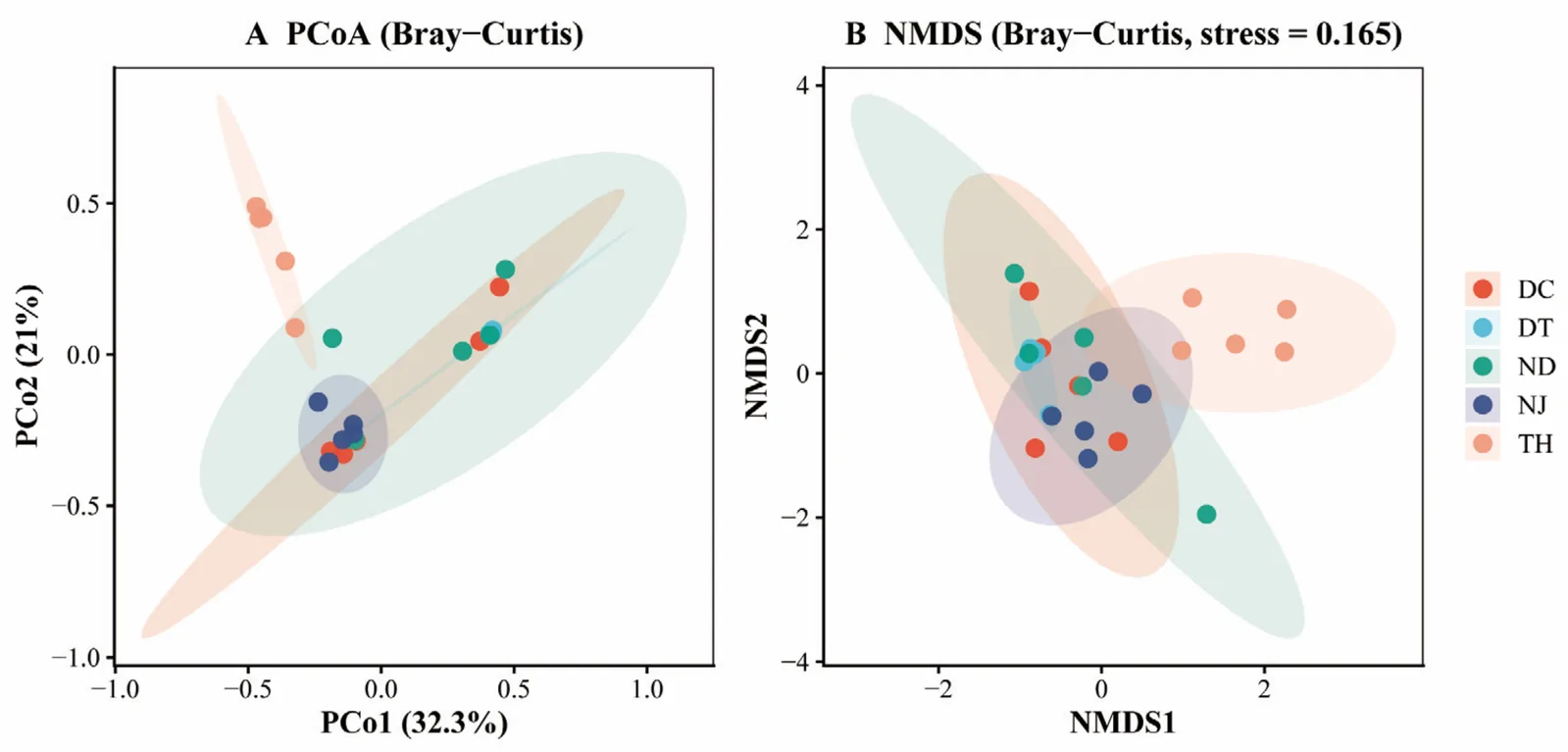
Beta diversity of gut microbial communities among the five aquaculture regions based on Bray–Curtis dissimilarity. (**A**) Principal coordinates analysis (PCoA). (**B**) Non-metric multidimensional scaling (NMDS; stress = 0.165). Points represent composite intestinal samples from each aquaculture region, and shaded ellipses indicate 95% confidence intervals. PERMDISP showed no significant difference in multivariate dispersion among cultured groups (F = 1.602, *p* = 0.199), whereas PERMANOVA detected a significant regional effect on gut microbial community composition (R^2^ = 0.414, F = 3.530, *p* = 0.001).

In this study, alpha and beta diversity were analysed to assess differences in intestinal microbial community structures among the 5 aquaculture regions. Alpha diversity analysis showed limited and index-specific variation among groups (Figure 2). The Shannon index differed significantly among the 5 aquaculture regions (*p =* 0.0384; Figure 2A). The Chao1 richness index showed no significant difference among regions (*p =* 0.6520; Figure 2B). The Simpson index also differed significantly among regions (*p =* 0.0121; Figure 2C), suggesting variation in community dominance or evenness. Pielou’s evenness index showed a significant difference among regions (*p =* 0.0257; Figure 2D). Together, variation was mainly associated with diversity and evenness-related changes rather than differences in overall richness. Beta diversity was evaluated using principal coordinate analysis (PCoA) and non-metric multidimensional scaling (NMDS). In PCoA (Figure 3A), PCo1 accounted for 32.3% of the variance and PCo2 accounted for 21.0%. The NMDS analysis showed a moderate ordination fit (stress = 0.165; Figure 3B). The microbial community structures of the five aquaculture regions showed partial overlap, but some degree of regional separation was also observed. PERMDISP analysis showed no significant difference in multivariate dispersion among the cultured groups (F = 1.602, *p* = 0.199), indicating that the PERMANOVA result was not primarily driven by differences in within-group dispersion. PERMANOVA based on Bray–Curtis distances detected a significant regional effect on gut microbial community composition (R^2^ = 0.414, F = 3.530, *p* = 0.001; Figure 3). This result suggests that gut microbial community composition differed among the five aquaculture regions, although the ordination patterns still showed considerable overlap among groups.

### 3.3. Microbial Community Structure and Predicted Phenotypes

**Figure 4:**
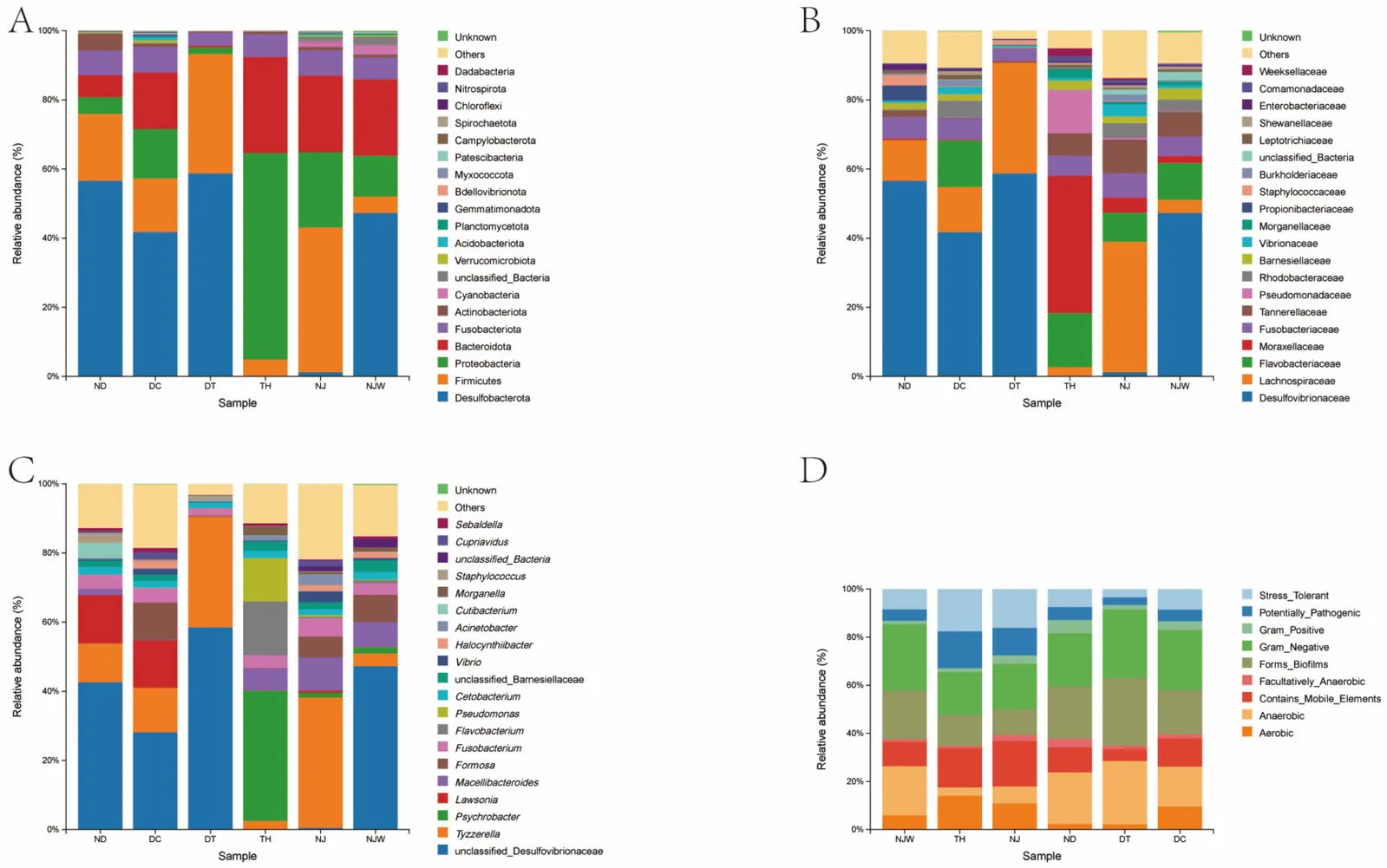
Taxonomic and functional composition of intestinal microbiota across sampling regions. (**A**) Relative abundance at the phylum level; (**B**) relative abundance at the family level; (**C**) relative abundance at the genus level; (**D**) BugBase-based prediction of microbial phenotypic characteristics.

In this study, the microbial community structures and predicted functional characteristics of different groups (NJW, NJ, DT, DC, ND, TH) were analysed, revealing differences in microbial composition and predicted phenotypic profiles among the groups. The phylum-level relative abundance analysis (Figure 4A) showed that *Proteobacteria*, *Bacteroidota*, *Desulfobacterita*, *Firmicutes*, and *Fusobacteria* were the predominant phyla in the intestinal microbiota of yellow croaker from different regions. Additionally, the relative abundance of *Desulfobacterita* was lower in the NJ group, and the relative abundance of Proteobacteria was lower in the DT group. At the family level, the intestinal microbiota of large yellow croaker exhibited distinct regional patterns (Figure 4B). *Desulfovibrionaceae* was the most dominant family across all groups, accounting for more than 40% of the community in ND, DC, DT, and NJW. In contrast, TH and NJ showed more diversified community structures, with *Moraxellaceae*, *Flavobacteriaceae*, and *Lachnospiraceae* contributing higher proportions. Specifically, ND and DC were characterized by high abundances of *Desulfovibrionaceae* and *Lachnospiraceae*, while DT exhibited co-dominance of *Desulfovibrionaceae* and *Vibrionaceae*. In the TH group, *Moraxellaceae* and *Pseudomonadaceae* were enriched, whereas NJ displayed relatively high proportions of *Flavobacteriaceae*, *Fusobacteriaceae*, and *Tannerellaceae*. The genus-level relative abundance analysis (Figure 4C) further revealed the dominance of genera such as *Tyzzerella* in the intestinal microbiota across different regions, highlighting differences in genus-level composition among the samples. BugBase-based prediction of microbial phenotypes (Figure 4D) indicated similar predicted phenotypic profiles of intestinal microbiota among the groups, with predicted aerobic and Gram-negative phenotypes accounting for relatively high proportions across samples.

### 3.4. Co-Occurrence Network Analysis of Microbial Communities

**Figure 5:**
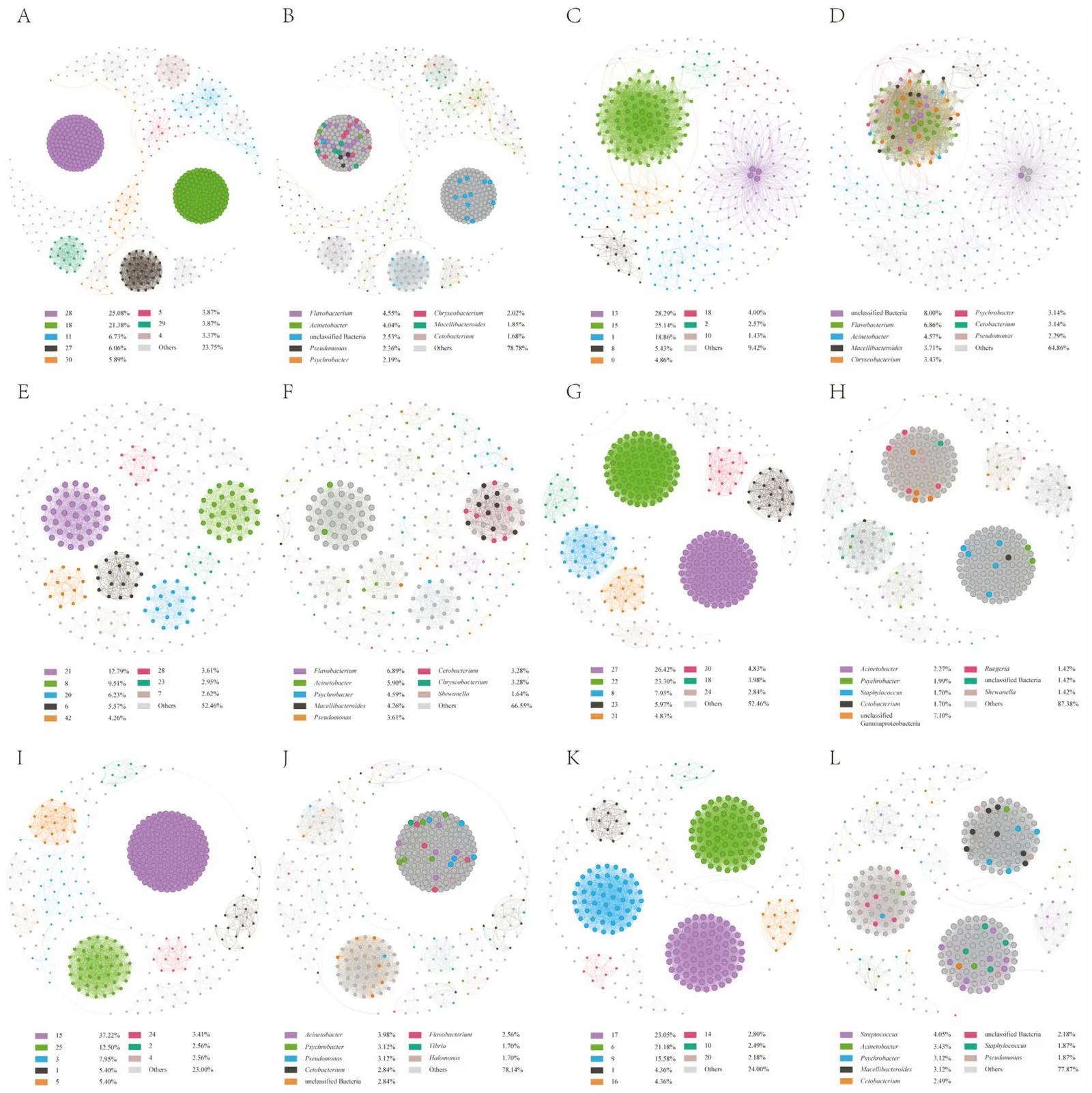
Intestinal microbial co-occurrence networks of *Larimichthys crocea*. Panels (**A**,**B**), (**C**,**D**), (**E**,**F**), (**G**,**H**), (**I**,**J**), and (**K**,**L**) represent NJ, NJW, TH, DC, DT, and ND, respectively. Panels (**A**,**C**,**E**,**G**,**I**,**K**) show network modules; the corresponding panels show genus-level taxonomy. Nodes represent OTUs, edges represent significant associations, and percentages indicate node proportions.

**Figure 6:**
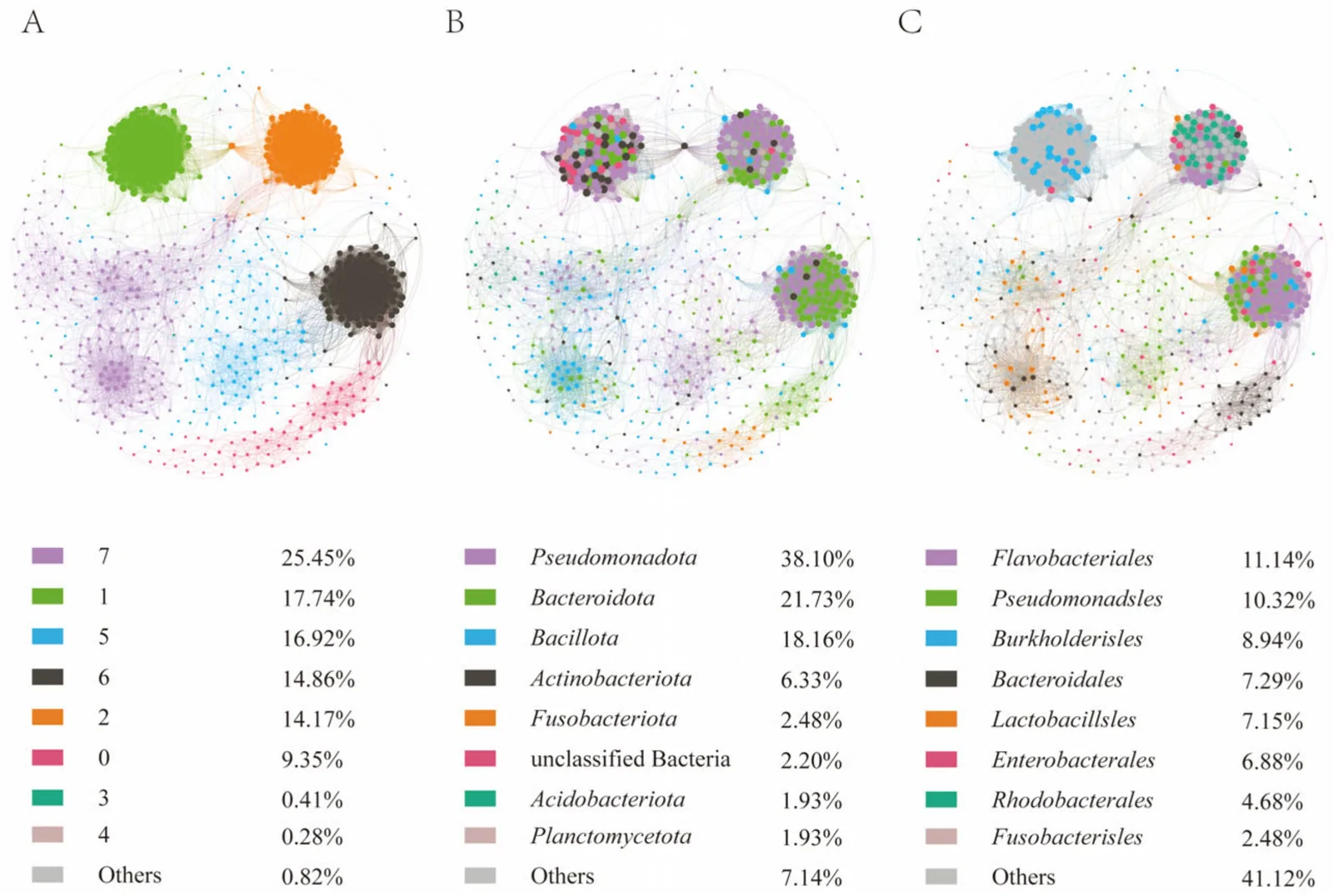
Overall intestinal microbial co-occurrence network of *Larimichthys crocea*. Panels (**A**–**C**) show the same network colored by module, phylum, and order, respectively. Nodes represent OTUs, edges represent significant associations, and percentages indicate node proportions.

**Figure 7:**
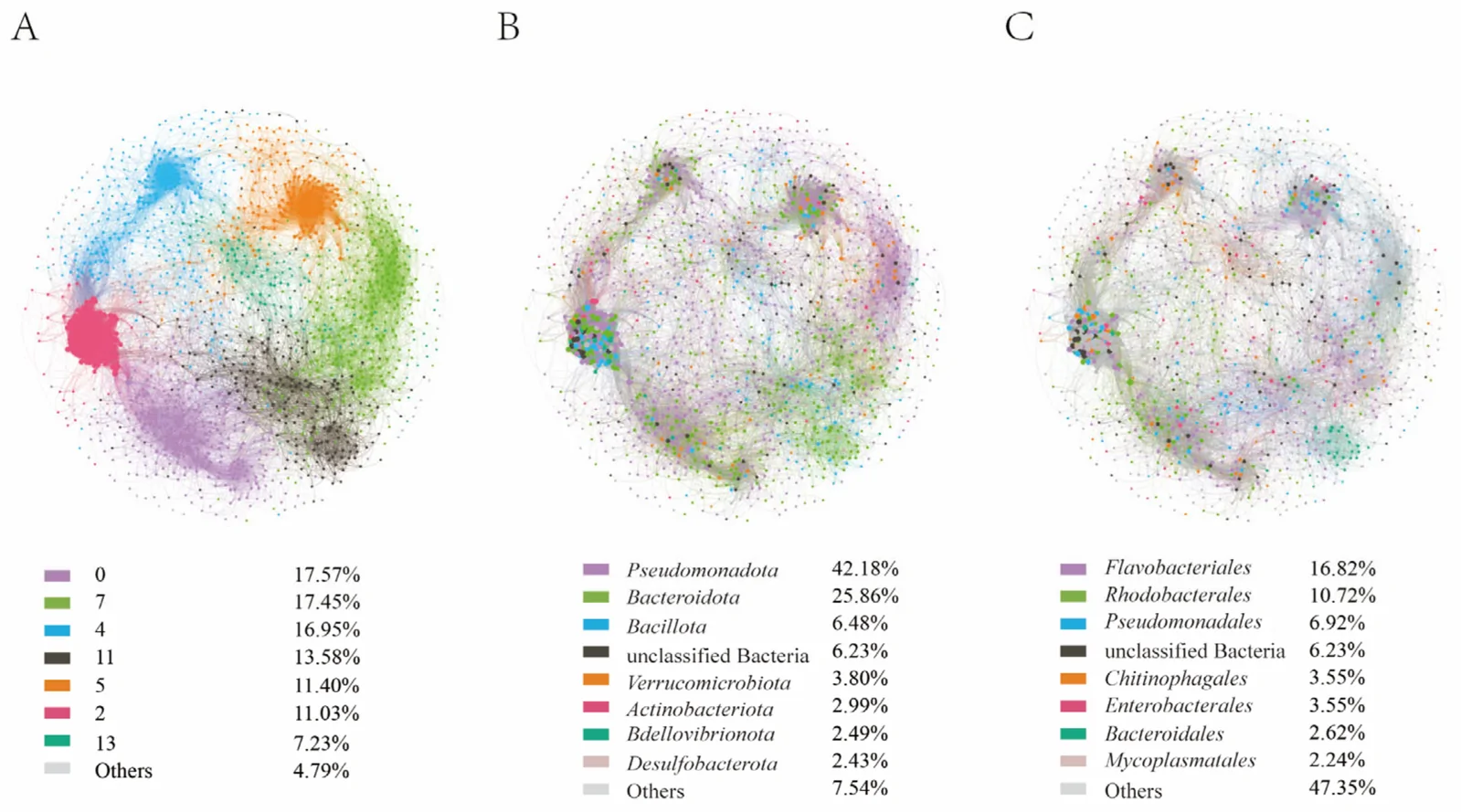
Overall microbial co-occurrence network of aquaculture water. Panels (**A**–**C**) show the same network colored by module, phylum, and order, respectively. Nodes represent OTUs, edges represent significant associations, and percentages indicate node proportions.

The interactions within bacterial communities were investigated by constructing Co-occurrence Network Analysis in the intestinal microbiota of each group. To achieve this, co-occurrence microbial networks were constructed for 2 microbial community datasets (the bacterial community in the aquaculture water of large yellow croaker and the intestinal microbiota of large yellow croaker), as well as 5 marine datasets. These marine regions included the Nanji Archipelago and Dongtou Archipelago near Wenzhou, the Zhoushan Archipelago, Dachen Archipelago near Taizhou, and the Ningde Sea area in Fujian (Figure 5, Figure 6 and Figure 7). The study utilized the Fruchterman–Reingold algorithm to construct co-occurrence networks for different groups, aiming to identify microbial interactions during the large yellow croaker aquaculture process. The key topological properties of these networks are presented in Table 1. While these differences are likely to reflect regional variation in microbial communities, potential confounding factors such as sequencing depth, compositionality, and zero-inflated data should also be considered. To minimize these effects, we rarefied all samples to the same sequencing depth and filtered OTUs absent in more than 50% of the samples prior to network construction.

By comparing network metrics, differences in network size, connectivity, density, and modular organization were observed between the water and intestinal microbial networks. The total number of nodes in the water microbial network (1605) was higher than that of the intestinal microbial network (727), and the total number of connections in the water microbial network (27,327) also exceeded that of the intestinal microbial network (18,187, Table 1). This indicates a larger scale and greater complexity in the water microbial network. However, the graph density (0.069) and average clustering coefficient (0.684) of the intestinal microbial network were higher than those of the water microbial network (graph density: 0.021; average clustering coefficient: 0.499, Table 1), indicating a more densely connected and locally clustered network structure in the intestinal microbiota. The modularity of the intestinal microbial network (0.725) was slightly higher than that of the water microbial network (0.716; Table 1), indicating a slightly stronger modular organization of microbial associations in the intestinal network. Additionally, the average weighted degree of the intestinal microbial network (39.602) was higher than that of the water microbial network (29.152; Table 1), indicating stronger overall weighted associations among connected nodes.

Although the average path length of the water microbial network (4.464) was slightly longer than that of the intestinal microbial network (4.209), the network diameters of the two were similar (12 and 13, respectively, Table 1), indicating minimal differences in the maximum path length. Network metrics also varied among regional groups. The NJ network had the highest total connections (20,844) and average degree (70.182, Table 1), demonstrating more complex node interactions. The TH network showed the highest modularity (0.802) and average clustering coefficient (1.000; Table 1), indicating a strongly modular and locally clustered network topology. The DT network had the highest graph density (0.163), while the NJW network had the lowest graph density (0.048) and average path length (4.510, Table 1), highlighting the diversity in microbial community structures. Overall, the water microbial network was characterized by a larger network size and a greater number of associations, whereas the intestinal microbial network exhibited higher density, clustering, and weighted connectivity. These results indicate distinct network topological patterns between water and intestinal microbial communities.

In the co-occurrence network analysis, six major modules were identified in the NJ intestinal microbiota (Figure 5A). Module 28 (25.08%) and Module 18 (21.38%) contained the largest proportions of network nodes, whereas Module 11 (6.73%), Module 27 (6.06%), and Module 30 (5.89%) accounted for smaller proportions. The remaining minor modules collectively represented 23.75% of the network. Most microbial associations occurred within rather than between modules, indicating a pronounced modular organization of the NJ network. At the taxonomic level, *Flavobacterium* (4.55%) and *Acinetobacter* (4.04%) were the most frequently represented genera among network nodes, followed by *Pseudomonas* (2.36%), *Psychrobacter* (2.19%), and *Chryseobacterium* (2.02%) (Figure 5B). *Macellibacteroides* (1.85%) and *Cetobacterium* (1.68%) represented smaller proportions, while unclassified bacteria accounted for 2.53%. The majority of network nodes were assigned to the “Others” category (78.78%), indicating that the NJ network comprised a taxonomically diverse set of genera, with no single genus accounting for a large proportion of the network.

In the modular composition of the NJW co-occurrence network, Module 13 (28.29%) and Module 15 (25.14%) contained the largest proportions of nodes, followed by Module 1 (18.86%). Module 8 (5.43%), Module 0 (4.86%), Module 18 (4.00%), Module 2 (2.57%), and Module 10 (1.43%) represented smaller proportions, while the remaining minor modules collectively accounted for 9.42% of the network nodes (Figure 5C). The network exhibited a clear modular structure, with relatively dense associations within modules and fewer connections between modules. At the genus level, unclassified Bacteria (8.00%) and *Flavobacterium* (6.86%) accounted for the largest proportions of network nodes, followed by *Acinetobacter* (4.57%), *Macellibacteroides* (3.71%), *Chryseobacterium* (3.43%), *Psychrobacter* (3.14%), *Cetobacterium* (3.14%), and *Pseudomonas* (2.29%) (Figure 5D). The majority of network nodes were assigned to the “Others” category (64.86%), indicating broad taxonomic representation beyond the most frequently represented genera.

In the co-occurrence network of the TH group, eight major modules were identified (Figure 5E). Among these, Module 21 (12.79%) contained the largest proportion of network nodes, followed by Module 8 (9.51%) and Module 20 (6.23%). Other modules with smaller node proportions included Module 6 (5.57%), Module 42 (4.26%), Module 28 (3.61%), Module 23 (2.95%), and Module 7 (2.62%), while the remaining minor modules collectively accounted for 52.46% of the network. The network displayed clear modularity, with relatively dense associations within modules and fewer connections between modules, indicating a pronounced modular organization of the TH network. At the genus level, *Flavobacterium* (6.89%) and *Acinetobacter* (5.90%) accounted for the largest proportions of network nodes, followed by *Psychrobacter* (4.59%), *Macellibacteroides* (4.26%), and *Pseudomonas* (3.61%) (Figure 5F). Other represented genera included *Cetobacterium* (3.28%), *Chryseobacterium* (3.28%), and *Shewanella* (1.64%), while 66.55% of the network nodes were assigned to the “Others” category. Overall, the taxonomic composition of the TH network was distributed across multiple genera, with several genera showing relatively higher representation among network nodes.

In the microbial co-occurrence network of the DC group, Module 27 (26.42%) contained the largest proportion of network nodes, followed by Module 22 (23.30%) and Module 8 (7.95%). Module 23 (5.97%), Module 21 (4.83%), Module 30 (4.83%), Module 18 (3.98%), and Module 24 (2.84%) accounted for smaller proportions (Figure 5G). The network showed a clear modular organization, with relatively dense associations within modules and fewer connections between modules. At the taxonomic level, unclassified Gammaproteobacteria (7.10%) accounted for the largest proportion of network nodes, followed by *Acinetobacter* (2.27%), *Psychrobacter* (1.99%), *Staphylococcus* (1.70%), and *Cetobacterium* (1.70%) (Figure 5H). Other represented taxa included *Ruegeria* (1.42%), unclassified Bacteria (1.42%), and *Shewanella* (1.42%), while 87.38% of the network nodes were assigned to the “Others” category. Overall, the taxonomic composition of the DC network was distributed across a broad range of taxa, with only a limited number of taxa showing relatively higher representation among network nodes.

In the intestinal microbiota co-occurrence network of the DT group, eight major modules were identified (Figure 5I). Among these, Module 15 (37.22%) contained the largest proportion of network nodes, followed by Module 25 (12.50%) and Module 3 (7.95%). Modules with smaller proportions included Module 1 (5.40%), Module 5 (5.40%), Module 24 (3.41%) and Module 2 (2.56%). The network exhibited a pronounced modular organization, with relatively dense associations within modules and fewer connections between modules. At the genus level, *Acinetobacter* (3.98%), *Psychrobacter* (3.12%), and *Pseudomonas* (3.12%) accounted for relatively large proportions of network nodes, followed by *Cetobacterium* (2.84%), unclassified Bacteria (2.84%), and *Flavobacterium* (2.56%) (Figure 5J). *Vibrio* (1.70%) and *Halomonas* (1.70%) accounted for smaller proportions, while 78.14% of the network nodes were assigned to the “Others” category. Overall, the DT network showed broad taxonomic representation, with several genera occurring at relatively higher frequencies among network nodes.

In the ND group, Module 17 (23.05%) and Module 6 (21.18%) contained the largest proportions of network nodes, followed by Module 9 (15.58%). Module 16 (4.36%), Module 1 (4.36%), Module 14 (2.80%), Module 10 (2.49%), and Module 20 (2.18%) accounted for smaller proportions, while the remaining modules collectively represented 24.00% of the network (Figure 5K). The network exhibited a distinct modular organization, with relatively dense associations within modules and fewer connections between modules. At the genus level, *Streptococcus* (4.05%), *Acinetobacter* (3.43%), and *Psychrobacter* (3.12%) accounted for relatively large proportions of network nodes, followed by *Macellibacteroides* (3.12%) and *Cetobacterium* (2.49%) (Figure 5L). Other represented taxa included unclassified Bacteria (2.18%), *Staphylococcus* (1.87%), and *Pseudomonas* (1.87%), while 77.87% of the network nodes were assigned to the “Others” category. Overall, the ND network showed broad taxonomic representation, with several genera occurring at relatively higher frequencies among network nodes.

**Figure 8:**
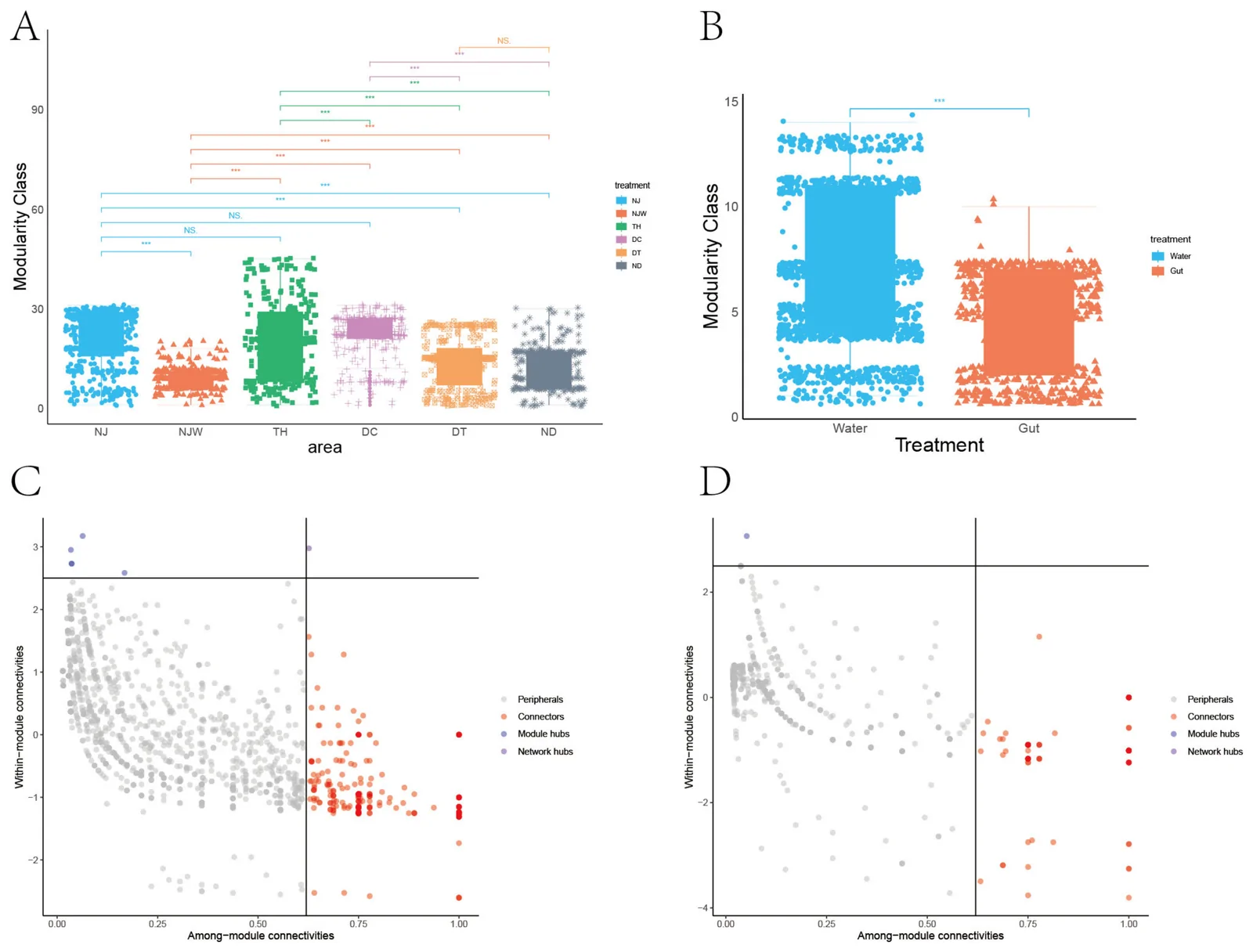
Network characteristics of gut and water microbiota in *Larimichthys crocea*. (**A**) Gut network modularity among sampling groups; (**B**) modularity comparison between gut and water networks; (**C**,**D**) Zi–Pi classification of node roles in water and gut networks, respectively. The horizontal and vertical solid lines indicate the classification thresholds of Zi = 2.5 and Pi = 0.62. *** indicates *p* < 0.001; NS indicates no significant difference.

Among the intestinal microbial networks of large yellow croaker groups, modularity varied across regions. The NJW network showed the lowest modularity value (Figure 8A, *p* < 0.05), indicating a comparatively weaker modular organization. In contrast, the TH network showed the highest modularity value (Figure 8A, *p* < 0.05, except for the comparison with NJ), indicating a comparatively stronger modular organization. No significant difference in modularity was detected between some groups, including NJ and DC (Figure 8A, *p* > 0.05). Overall, these results indicate regional variation in the modular organization of intestinal microbial association networks.

In this study, co-occurrence networks were constructed and analyzed for both aquaculture-water and intestinal microbial communities. In the aquaculture-water network, multiple modules were identified, with Module 0 (17.57%) and Module 7 (17.45%) containing the largest proportions of network nodes, followed by Module 4 (16.95%). Module 11 (13.58%), Module 5 (11.40%), and Module 2 (11.03%) also accounted for substantial proportions, whereas Module 13 (7.23%) and the remaining smaller modules collectively represented 7.23% and 4.79% of the nodes, respectively (Figure 6A). Modules were distinguished by different colours for visualization. The network showed relatively dense associations within modules and fewer connections between modules, indicating a pronounced modular organization.

At the phylum level, Proteobacteria accounted for the largest proportion of network nodes (42.18%), followed by Bacteroidota (25.86%) (Figure 6B). Firmicutes (6.48%) and unclassified bacteria (6.23%) also represented substantial proportions, whereas Verrucomicrobiota (3.80%), Actinobacteriota (2.99%), and Bdellovibrionota (2.49%) accounted for smaller proportions. Thus, Proteobacteria and Bacteroidota were the most frequently represented phyla among nodes in the aquaculture-water co-occurrence network.

At the order level, Flavobacteriales (16.82%) accounted for the largest proportion of network nodes, followed by Rhodobacterales (10.72%) and Pseudomonadales (6.92%) (Figure 6C). Unclassified bacteria (6.23%) and Chitinophagales (3.55%) also represented notable proportions, while other orders collectively accounted for 47.35%. Overall, the taxonomic composition of the aquaculture-water network was distributed across multiple bacterial orders, with Flavobacteriales and Rhodobacterales showing relatively higher representation among network nodes.

Similarly, the intestinal microbiota of farmed large yellow croaker was analyzed using microbial co-occurrence networks. Figure 7A illustrates the modular structure of the bacterial association network, which comprised multiple modules. Module 7 (25.45%) contained the largest proportion of network nodes, followed by Module 1 (17.74%) and Module 5 (16.92%). Module 6 (14.86%) and Module 2 (14.17%) also accounted for substantial proportions, whereas Module 0 (9.35%) and the remaining smaller modules represented lower proportions. Modules were distinguished by different colours for visualization. The network showed relatively dense associations within modules and fewer connections between modules, indicating a pronounced modular organization.

Figure 7B shows the phylum-level taxonomic composition of the intestinal microbial network. Proteobacteria accounted for the largest proportion of network nodes (38.10%), followed by Bacteroidota (21.73%) and Firmicutes (18.16%). Actinobacteriota (6.33%) and Fusobacteriota (2.48%) represented smaller proportions. Overall, Proteobacteria and Bacteroidota were the most frequently represented phyla among nodes in the intestinal microbial co-occurrence network.

Figure 7C further shows the order-level taxonomic composition of the network nodes. Flavobacteriales (11.14%) accounted for the largest proportion, followed by Pseudomonadales (10.32%) and Burkholderiales (8.94%). Bacteroidales (7.29%) and Lactobacillales (7.15%) also represented notable proportions, whereas Enterobacterales (6.88%) and Rhodobacterales (4.68%) accounted for smaller proportions. Overall, the intestinal microbial network contained a broad taxonomic representation at the order level, with Flavobacteriales, Pseudomonadales, and Burkholderiales showing relatively higher representation among network nodes.

The modularity values differed between the aquaculture-water and intestinal microbial networks (Figure 8B), indicating differences in their modular organization. The Zi–Pi analysis further characterized the topological roles of nodes within the two networks. In the aquaculture-water network, most nodes were classified as peripherals (Zi < 2.5, Pi < 0.62), indicating that their associations were predominantly confined to their respective modules (Figure 8C). Connectors, module hubs, and network hubs were also identified (Figure 8C and Table 2), representing nodes with different patterns of within- and among-module connectivity. In particular, connectors exhibited relatively high participation across modules, whereas module hubs showed high within-module connectivity. Similarly, most nodes in the intestinal microbial network were classified as peripherals (Figure 8D and Table 3), while fewer nodes were assigned to connector and hub categories. These results indicate differences between the aquaculture-water and intestinal networks in the distribution of node topological roles and patterns of within- and among-module associations. Because the present co-occurrence analysis is based on statistical associations, these topological differences were not interpreted as direct evidence of microbial functional specialization or ecological interactions.

Additionally, SourceTracker analysis revealed that the majority of gut microbiota across individuals was assigned to the “unknown” category, indicating that their origins could not be fully explained by the available water samples (Figure S1). Minor contributions from local water environments were detected, with the DT and ND sites showing slightly higher proportions compared to other regions. These findings suggest that while aquaculture water may influence gut microbiota composition to some extent, a substantial fraction of intestinal microbes originates from uncharacterized or host-associated sources.

**Figure 9:**
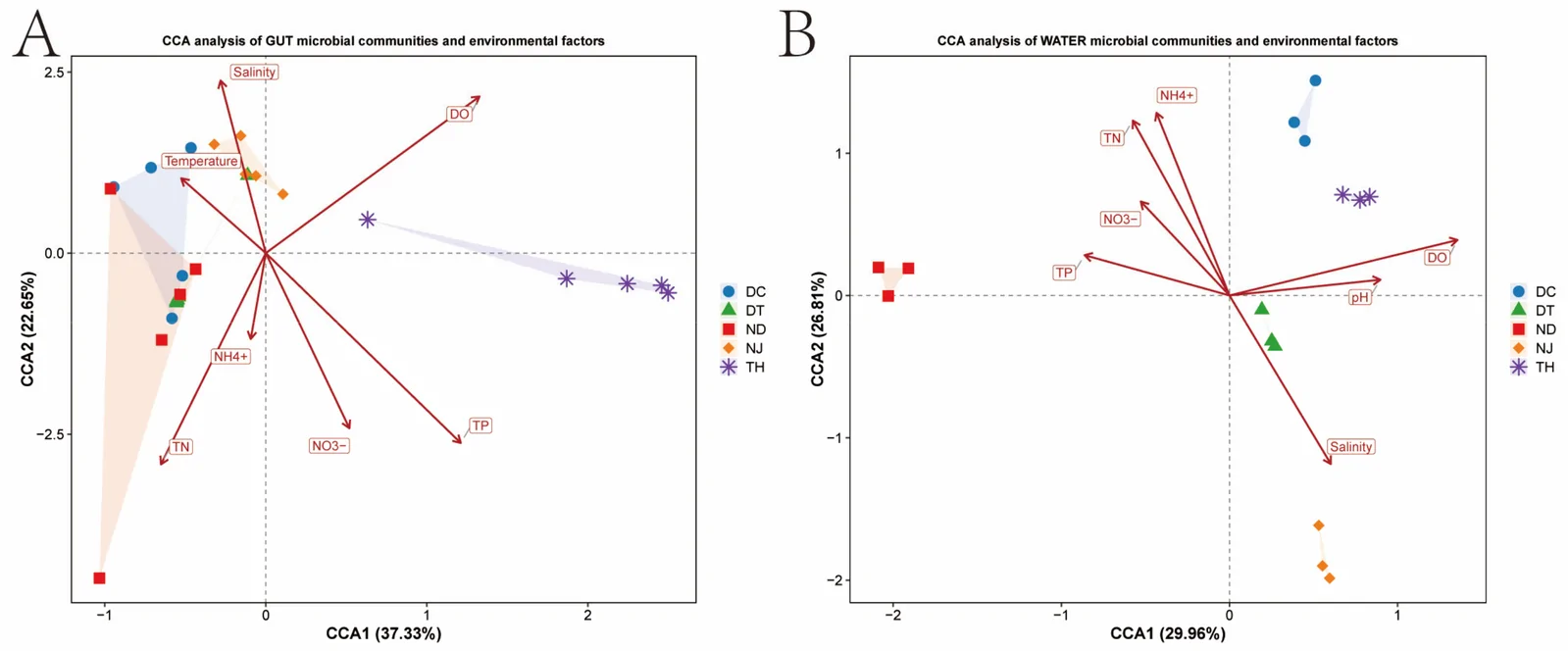
CCA of gut and water microbial communities and environmental variables. (**A**) Gut microbiota; (**B**) water microbiota. Colors and shapes indicate sampling groups, and shaded areas represent group distributions. Red arrows indicate environmental variables. Axis percentages represent the proportions of constrained variation explained by CCA1 and CCA2.

Canonical correspondence analysis (CCA) was further performed to explore the associations between environmental factors and microbial community structures (Figure 9 and Table S2). For gut microbial communities, the first two CCA axes explained 37.33% and 22.65% of the constrained variation, respectively (Figure 9A). The TH samples were mainly distributed along the positive direction of CCA1, whereas the DC, DT, ND, and NJ samples were primarily located on the negative side of CCA1. Environmental vectors, including dissolved oxygen, salinity, temperature, ammonium, total nitrogen, nitrate, and total phosphorus, contributed to the ordination pattern, suggesting that regional environmental conditions may be associated with gut microbial community variation. For water microbial communities, CCA1 and CCA2 explained 29.96% and 26.81% of the constrained variation, respectively (Figure 9B). Compared with gut microbial communities, water microbial communities showed clearer regional separation. The NJ water samples were mainly associated with the salinity gradient, whereas TH samples were positioned closer to the directions of dissolved oxygen and pH. Nutrient-related variables, including total nitrogen, nitrate, ammonium, and total phosphorus, also contributed to the distribution of water microbial communities. Overall, these results indicate that environmental heterogeneity among aquaculture regions was associated with both water and gut microbial community structures, but the association appeared stronger for water microbiota than for gut microbiota.

Overall, these results indicate that environmental heterogeneity among aquaculture regions was associated with both water and gut microbial community structures, with a stronger association for water microbiota than for gut microbiota, consistent with the subsequent variation-partitioning analysis (Table S2).

## 4. Discussion

The results of this study showed that the intestinal microbiota of large yellow croaker shared several dominant bacterial groups across regions, while also exhibiting moderate and group-specific compositional variation. *Proteobacteria*, *Bacteroidota*, and *Firmicutes* were the dominant phyla in most samples, with Proteobacteria being the most abundant. The predominance of these phyla suggests that they may represent important members of the gut microbial community of *L. crocea*, potentially contributing to nutrient metabolism, carbon utilization, and host adaptation [6,19,31]. However, the relative abundance of dominant taxa varied among regions. For example, fish from Dongtou (DT) and Ningde (ND) showed a lower relative abundance of *Proteobacteria*, whereas fish from the Nanji aquaculture group (NJ) showed relatively higher proportions of Firmicutes and several group-specific dominant genera. At the genus level, *Cetobacterium* was enriched in DT and was also abundant in ND and Taizhou/Dachen (DC), together with notable proportions of *Psychrobacter*, *Lawsonia*, and *Macellibacteroides*. In contrast, *Tyzzerella* showed a relatively higher abundance in NJ. Previous studies have suggested that *Tyzzerella* may be associated with specific dietary backgrounds, including herbivorous or plant-enriched dietary regimes in fish [32,33,34]. Therefore, the higher abundance of *Tyzzerella* in NJ may be related to local dietary resources or feeding environments, although its functional role in large yellow croaker requires further validation.

Alpha-diversity analysis further suggested that the differences among groups were limited and index-specific. Shannon diversity differed significantly among regions, whereas Chao1 richness showed no significant difference, indicating that the observed variation was not mainly associated with overall richness. In contrast, both the Simpson index and Pielou’s evenness index showed significant variation, suggesting that regional differences in alpha diversity were more closely related to community diversity, dominance, and evenness. Therefore, the regional patterns observed in this study should not be interpreted as a uniform shift in all aspects of gut microbial diversity. Instead, they may mainly reflect changes in community evenness, dominance structure, and the relative abundance of specific bacterial groups. Such compositional variation may be associated with multiple factors, including local environmental conditions, aquaculture system, stocking density, feed formulation, host body size, and sex-related physiological differences [35,36,37,38,39]. In the present study, aquacultured fish populations with broadly comparable farming conditions were preferentially selected to reduce the influence of management-related differences. However, because this study was conducted across multiple farming regions, some background factors, such as minor differences in feeding practices and variation in body size among certain groups, could not be fully controlled. These factors may have contributed to part of the unexplained variation in gut microbial communities. Therefore, the observed regional differences should be interpreted as patterns of microbial community variation under regional farming conditions, rather than as causal effects directly attributable solely to the geographic region itself.

Beta-diversity analyses based on PCoA, NMDS, and PERMANOVA further revealed regional variation in the gut microbial communities of farmed large yellow croaker. Although microbial communities partially overlapped among most aquaculture regions, some degree of separation was observed between specific groups, such as DT and NJ. PERMANOVA detected a significant regional effect, with aquaculture region explaining 41.4% of the total variation in gut microbial composition (R^2^ = 0.414, F = 3.530, *p* = 0.001). Moreover, the nonsignificant PERMDISP result indicated that this regional differentiation was not primarily driven by differences in within-group dispersion. These findings suggest that aquaculture region was an important source of gut microbial variation, although considerable variation remained within regions. The partial similarity among groups may be related to the shared host species, broadly comparable aquaculture backgrounds, and the presence of common dominant bacterial groups, including *Proteobacteria*, *Bacteroidota*, and *Firmicutes* [6,40,41]. Nevertheless, the aquaculture region represents a composite factor encompassing local environmental conditions, farming systems, feeding practices, microbial exposure, and host-related backgrounds, rather than a purely geographical variable. Therefore, the observed community differentiation should not be attributed to geographical location alone. In addition, some background differences among groups could not be completely controlled under field farming conditions and may have contributed to both the regional differentiation and the residual variation in gut microbial community composition [38].

The differences observed among NJ, DT, and ND may also reflect the combined influence of regional aquaculture settings and environmental backgrounds. The NJ group showed relatively higher microbial diversity and a more complex community profile, which may be related to the open-ocean hydrodynamic conditions and local feeding environment of the Nanji Islands [42,43]. However, this pattern should not be directly interpreted as evidence of a healthier or more stable gut microbiota without additional functional validation. The relatively dispersed microbial patterns observed in the wild group may reflect greater individual heterogeneity and natural dietary variation under wild conditions, but this comparison should be interpreted cautiously because the wild individuals were limited in number and were analyzed individually rather than as pooled samples [35,36,37,43]. By contrast, ND was collected from a fish-raft culture system in a relatively enclosed aquaculture setting, where differences in water exchange and farming intensity may be associated with microbial exposure and community composition [24]. DT represents a cage culture setting with stronger hydrodynamic exchange, which may dilute environmental microbial inputs while imposing different environmental selection pressures [44]. Collectively, these results suggest that regional gut microbiota patterns in large yellow croaker may reflect the combined effects of environmental conditions, aquaculture practices, feeding background, host-related factors, and microbial selection processes, rather than by geographical location alone.

Beyond taxonomic composition and alpha diversity, co-occurrence network analysis was further used to explore potential microbial association patterns in the gut microbiota of *L. crocea* [45]. Differences in network topology were observed among regions, suggesting that regional populations may differ not only in taxonomic composition but also in potential microbial association structure [46]. The co-occurrence network of fish from the Nanji aquaculture group (NJ) showed relatively high connectivity and average node degree, indicating more frequent potential associations among microbial taxa [47,48]. Highly connected microbial networks are often considered to have greater interaction complexity and may be associated with functional redundancy and resilience under environmental fluctuations [48,49]. However, because co-occurrence networks are based on statistical associations rather than direct experimental evidence, these results should be interpreted as potential microbial interaction patterns rather than confirmed functional relationships [50,51]. In contrast, the Dongtou group (DT) had more network modules but lower graph density and weaker inter-module connectivity, suggesting a more fragmented association pattern among microbial taxa [52,53]. This may indicate weaker coordination among microbial groups, although the functional consequences of this network structure require further validation.

Network modularity also differed among regions, suggesting variability in microbial community organization and potential functional compartmentalization [48,54]. For example, the Zhoushan population (TH) showed the highest modularity, which may indicate clearer subdivision of microbial associations within the gut microbial network. Such modular organization could be related to regional farming conditions or environmental stability, but it should not be interpreted as direct evidence of mature functional modules without metagenomic or experimental validation [55]. By contrast, the wild *L. crocea* group (NJW) showed the lowest modularity, suggesting a less compartmentalized association structure and weaker clustering among microbial taxa [53]. Previous studies on Atlantic salmon have also reported differences in microbiome composition and network organization between wild and captive populations, indicating that rearing environment can influence host-associated microbial community structure [40,56]. In the present study, the difference between wild and cultured *L. crocea* appeared to be more evident in network topology. However, this interpretation should be treated cautiously because the wild individuals were limited in number and were analyzed individually, whereas cultured samples were pooled. Overall, regional differences in gut microbial co-occurrence networks may reflect the combined effects of microbial association strength, environmental conditions, aquaculture practices, feeding background, and host-driven microbial selection. These network results provide an additional ecological perspective for understanding regional gut microbiota patterns, but they do not by themselves demonstrate functional differences among regions.

In addition to gut microbiota networks, this study compared the co-occurrence network structure of microbial communities in aquaculture water. The water microbial network contained more nodes, more total connections, and a higher average node degree than the gut microbial network, suggesting greater structural complexity and broader potential associations among environmental microbial taxa [57,58,59]. This more connected structure may be associated with environmental heterogeneity, including nutrient levels, hydrodynamic disturbance, temperature variation, and other water quality factors [60,61]. In contrast, although the intestinal microbial network was smaller in scale, it showed higher graph density and clustering coefficients, suggesting a more compact association structure within the host gut environment. This pattern suggests that the gut microbial community showed greater compositional similarity across regions than the surrounding water microbiota. Previous studies have suggested that host physiology, immune status, diet, and intestinal conditions may influence gut microbial composition [62,63,64]; however, these potential mechanisms were not directly assessed in the present study.

The modularity pattern and Zi–Pi analysis further indicated differences between water and gut microbial networks. The aquaculture water network contained more connectors and module hubs, whereas the gut network was mainly composed of peripheral nodes. This suggests that environmental microbial communities may contain more taxa linking different modules, while gut microbial communities may be more locally organized within specific modules [65,66]. However, the relationship between water and gut microbiota should be interpreted cautiously. The gut microbial community should not be regarded as simply originating from aquaculture water. Rather, the surrounding water microbiota may represent one potential source contributing to gut microbial assembly, while feed, sediment, biofilms, aquaculture facilities, and host-associated selection processes may also contribute substantially [67]. Therefore, the comparison between water and gut networks suggests a potential connection between environmental microbial exposure and intestinal microbial organization, but also highlights the strong filtering role of the host gut environment. Taken together, these findings suggest that environmental factors, host physiology, aquaculture practices, and microbial association patterns jointly contribute to shaping the gut microbial community structure of *L. crocea*.

In summary, the gut microbiota of farmed *L. crocea* exhibited both shared characteristics and regional distinctions in taxonomic composition and network organization. *Proteobacteria*, *Bacteroidota*, and *Firmicutes* were consistently dominant across most regional samples, suggesting that these phyla may represent important members of a membership-based core microbiota in *L. crocea* [41,68]. Here, the core microbiota refers to bacterial taxa that were repeatedly detected across different groups, rather than taxa that were necessarily functionally identical across all individuals. Such shared bacterial groups may contribute to basic intestinal functions, including nutrient metabolism, intestinal homeostasis, and host–microbe interactions, but their specific functional roles require further validation by metagenomic, transcriptomic, or experimental approaches [69,70,71,72,73]. Meanwhile, regional differences were observed in the relative abundance of dominant taxa, index-specific alpha-diversity patterns, and network topology. These patterns may reflect the combined influence of regional aquaculture environments, microbial inputs from surrounding waters, feeding regimes, farm management practices, and host-related factors [70,74,75]. Therefore, the gut microbiota of large yellow croaker should be interpreted as a shared but variable microbial system shaped by both common host-associated features and region-specific environmental or farming backgrounds.

From an aquaculture perspective, regional variation in gut microbial composition, predicted functional characteristics, and network organization may provide useful supplementary information for understanding fish–environment relationships in farmed large yellow croaker. However, a more compact or highly modular microbial network should not be directly interpreted as evidence of improved fish health, disease resistance, or production performance without supporting physiological and functional measurements [63,76,77]. Likewise, regions with relatively lower microbial diversity or less connected network structures should not be assumed to represent poorer health conditions solely on the basis of microbiota data. Instead, microbiota-based assessments should be integrated with water quality parameters, feeding records, growth performance, host physiological and immune indicators, and disease status. The regional patterns identified in this study therefore provide a baseline for future investigations linking gut microbial structure and predicted functional potential with host performance under different aquaculture conditions. Although feed optimization, probiotic supplementation, and microbiota-oriented management may represent potential applications, their effectiveness should be evaluated through controlled experiments before region-specific management recommendations are proposed. Overall, these findings highlight the potential value of gut microbiota as a complementary indicator in aquaculture research, while emphasizing that environmental, farming-related, and host-related variables must be jointly considered when interpreting regional microbial differences.

## 5. Conclusions

This study demonstrated that farmed large yellow croaker (*Larimichthys crocea*) maintained a broadly shared gut microbial community structure across different aquaculture regions while exhibiting clear region-associated variation in microbial diversity, taxonomic composition, predicted functional characteristics, and network organization. *Proteobacteria*, *Bacteroidota*, and *Firmicutes* were consistently dominant across regions, indicating the presence of common bacterial groups potentially involved in fundamental intestinal processes. Nevertheless, the observed regional variation in gut microbial composition may reflect the combined influence of multiple environmental, host-related, and aquaculture-management factors. In addition to the measured environmental variables, unmeasured differences in feed composition, stocking density, fish age and body condition, water exchange, disease history, antibiotic use, and other local management practices may also contribute to the observed patterns. These factors were not systematically evaluated in the present study and should therefore be considered when interpreting regional differences in gut microbiota. The predicted functional profiles were comparatively conserved across regions despite differences in taxonomic composition, suggesting that regional taxonomic turnover may not necessarily result in equivalent changes in broad microbial functional potential. Comparisons between aquaculture water and gut microbiota further indicated that local water represented a detectable but limited potential source of intestinal microorganisms, whereas the host gut environment exerted substantial selective filtering on microbial community assembly. Overall, these findings provide a regional baseline for understanding the structure and predicted functional characteristics of the gut microbiota in farmed large yellow croaker and support future studies integrating microbial data with environmental conditions, farming practices, and host physiological performance.

## Figures and Tables

**Table 1:** Network Metrics for Microbial Communities in Water and Intestine.

Metric	NJ	NJW	DT	DC	TH	ND	Intestinal Microbiota	Water Microbiota
Total nodes	594	350	352	352	305	321	727	1605
Total links	20,844	2910	10,046	8704	1981	6586	18,187	27,327
Average Degree	70.182	16.629	57.08	49.455	12.99	41.034	50.033	34.052
Average Weighted Degree	70.154	15.583	57.062	49.455	12.99	41.034	39.602	29.152
Network Diameter	12	17	10	1	1	1	13	12
Graph Density	0.118	0.048	0.163	0.141	0.043	0.128	0.069	0.021
Connected Components	32	17	27	32	46	31	5	7
Modularity	0.571	0.41	0.272	0.61	0.802	0.677	0.725	0.716
Average Clustering Coefficient	0.909	0.463	0.884	1	1	1	0.684	0.499
Average Path Length	1.184	4.51	1.123	1	1	1	4.209	4.464

**Table 2:** Network Roles and Key Microbial Taxa in Water Microbiota.

Phylum	Order	Degree	Modularity Class	Type
*Proteobacteria*	*Enterobacterales*	62	11	Module hubs
*Bacteroidota*	*Bacteroidales*	58	11	Module hubs
*Bacteroidota*	*Flavobacteriales*	57	11	Module hubs
*Bacteroidota*	*Bacteroidales*	55	11	Module hubs
*Bacteroidota*	*Bacteroidales*	55	11	Module hubs
*Bacteroidota*	*Flavobacteriales*	54	7	Connectors
*Desulfobacterota*	*Bradymonadales*	47	11	Connectors
*Proteobacteria*	*Pseudomonadales*	43	11	Connectors
*Proteobacteria*	*Enterobacterales*	43	11	Connectors
*Bacteroidota*	*Flavobacteriales*	34	7	Connectors
*Bacteroidota*	*Flavobacteriales*	33	5	Connectors
*Proteobacteria*	*Pseudomonadales*	29	1	Network hubs
*Bacteroidota*	*Flavobacteriales*	29	7	Connectors
*Bacteroidota*	*Sphingobacteriales*	29	7	Connectors
*Bacteroidota*	*Flavobacteriales*	27	5	Connectors
*Desulfobacterota*	*Desulfobulbales*	27	1	Connectors
*unclassified_Bacteria*	*unclassified_Bacteria*	24	11	Connectors
*Bdellovibrionota*	*Bdellovibrionales*	23	11	Connectors
*Bacteroidota*	*Cytophagales*	22	1	Connectors
*Proteobacteria*	*Coxiellales*	22	4	Connectors

**Table 3:** Network Roles and Key Microbial Taxa in Intestinal Microbiota.

Phylum	Order	Degree	Modularity Class	Type
*Proteobacteria*	*Pseudomonadales*	38	5	Module hubs
*Cyanobacteria*	*Cyanobacteriales*	12	2	Connectors
*Firmicutes*	*Erysipelotrichales*	10	5	Connectors
*Proteobacteria*	*Rhodobacterales*	7	7	Connectors
*Firmicutes*	*Lachnospirales*	7	5	Connectors
*Proteobacteria*	*Burkholderiales*	6	7	Connectors
*Firmicutes*	*Oscillospirales*	6	5	Connectors
*Firmicutes*	*Lactobacillales*	6	5	Connectors
*Bacteroidota*	*Bacteroidales*	5	5	Connectors
*Actinobacteriota*	*Propionibacteriales*	5	6	Connectors
*Bacteroidota*	*Flavobacteriales*	4	1	Connectors
*Proteobacteria*	*Enterobacterales*	4	5	Connectors
*Proteobacteria*	*Burkholderiales*	4	7	Connectors
*Proteobacteria*	*Enterobacterales*	4	6	Connectors
*Proteobacteria*	*Enterobacterales*	4	1	Connectors
*Proteobacteria*	*Enterobacterales*	3	4	Connectors
*Firmicutes*	*Erysipelotrichales*	3	7	Connectors
*Bacteroidota*	*Bacteroidales*	3	5	Connectors
*Proteobacteria*	*Pseudomonadales*	3	7	Connectors
*Actinobacteriota*	*Propionibacteriales*	3	5	Connectors

## Data Availability

The datasets generated and/or analyzed during the current study are not publicly available due to ongoing related research but are available from the corresponding author upon reasonable request.

## Supplementary Materials

The following supporting information can be downloaded at https://www.mdpi.com/article/10.3390/biology15171463/s1. Table S1: Environmental factors and body trait data of *Larimichthys crocea*; Figure S1: SourceTracker analysis of potential microbial source contributions to the gut microbiota of *Larimichthys crocea*; Table S2: Permutation test results of canonical correspondence analysis (CCA) for associations between environmental factors and microbial community composition.
